# Type I-F CRISPR-associated transposons contribute to genomic plasticity in Shewanella and mediate efficient programmable DNA integration

**DOI:** 10.1099/mgen.0.001476

**Published:** 2025-08-19

**Authors:** Xinlei Wang, Zhijie Chen, Cong Liu, Ziyang Zhang, Yijun Deng, Linfang Tao, James M. Tiedje, Jie Deng

**Affiliations:** 1 Zhejiang Tiantong Forest Ecosystem National Observation and Research Station, School of Ecological and Environmental Sciences, East China Normal University, Shanghai, PR China; 2 Center for Global Change and Ecological Forecasting, Institute of Eco-Chongming, East China Normal University, Shanghai, PR China; 3 Center for Microbial Ecology, Department of Plant, Soil and Microbial Sciences, Michigan State University, East Lansing, MI 48824, USA

**Keywords:** CRISPR-associated transposon (CAST), genome evolution, horizontal gene transfer (HGT), mobile genetic element (MGE), *Shewanella*

## Abstract

The genome plasticity of species and strains in the genus *Shewanella* is closely associated with the diverse mobile genetic elements embedded in its genomes. One mobile element with potential for accurate and efficient DNA insertion in *Shewanella* is the type I-F3 CRISPR-associated transposon (I-F3 CAST). However, relatively little is known about the distribution and ecological significance of I-F3 CASTs and whether they could be suitable as a tool for targeted genetic manipulation *in situ*. To better understand the distribution of I-F3 CASTs in *Shewanella*, we analysed 602 *Shewanella* genomes. We found that I-F3 CASTs were present in 12% of all genomes, although differences in both gene arrangement and integration locus were observed. These *Shewanella* I-F3 CASTs carried up to 89 cargo genes, which were associated with diverse functions, including defence, resistance and electron transfer, demonstrating an important role in genomic diversification and ecological adaptation. We tested whether the I-F3 CAST present in *Shewanella* sp. ANA-3 enhanced gene insertion, both *in situ* and in a heterologous host. We observed I-F3 CAST-mediated crRNA-targeted integration of the supplied genes into the *pyrF* locus in *Shewanella* sp. ANA-3. Heterologous gene insertion with high integration efficiency in *Escherichia coli* was also demonstrated using a simplified version of ANA-3 I-F3 CAST. Altogether, this work highlights the important role of I-F3 CASTs in promoting genomic plasticity of the *Shewanella* genus and demonstrates the gene-editing capability of ANA-3-CAST both endogenously and heterologously.

## Graphical Abstract

Comparative genomic analysis revealed that I-F3 CASTs serve as important mobile genetic elements mediating horizontal transfer of functional genes associated with defence, resistance and electron transfer in *Shewanella*. The transposition function of a new I-F3 CAST variant carried by strain ANA-3 was assessed both endogenously and heterologously.

**Impact Statement**

As a newly discovered type of mobile genetic element, the CAST catalyses flexible transposition of DNA fragments into chromosomes and plasmids of prokaryotes. *Shewanella* is an environmentally important bacterial genus and mediates bioremediation and biogeochemical cycling of multiple elements. In this study, we characterized the diversity of CASTs present in *Shewanella* genomes, demonstrating the presence of cargo genes related to stress resistance, virulence and auxiliary metabolism, which play important roles in environmental adaptation and metabolic versatility. We further characterized the CAST present in *Shewanella* sp. ANA-3, identifying minimal components needed for efficient and precise DNA insertion in heterologous hosts. This newly developed tool provides an alternative to existing tools that use large plasmids and/or multi-plasmid systems, which limits their use for genome editing in non-model strains. Collectively, the findings of this study not only revealed the role of CASTs in shaping bacterial genome plasticity but also broadened the CAST-based gene-editing toolset.

## Data Summary

Only publicly available genome data from NCBI were used for this research. The authors confirm that all supporting data, code and protocols have been provided within the article or through supplementary data files.

## Introduction

The type I-F CRISPR-associated transposon (CAST) is a type of transposable element originally characterized in *Gammaproteobacteria* [1]. It mediates horizontal gene transfer of a diverse range of cargo genes, including those related to antibiotic and heavy metal resistance and even central carbon metabolism [2,3]. Functionally, the CAST catalyses efficient and seamless integration using transposase proteins similar to those encoded by Tn7 transposons; however, a key mechanistic difference from Tn7 transposons is that the transposase proteins coordinate with nuclease-deficient CRISPR-Cas system proteins to facilitate RNA-guided DNA transposition [4,6]. Currently, six types of CRISPR-Cas systems associated with Tn7-like transposons have been discovered: types I-B, I-C, I-D, I-F, IV and V [7,8]. Among these, type I-B appears to have been captured by two different transposons through separate evolutionary events, leading to its classification into I-B1 and I-B2 [9]. Type I-B, I-D, I-F and V-K CASTs have all been experimentally validated to mediate transposition [5,6, 10]. Transposition functions of type I-C and IV CASTs have been proposed but not experimentally validated. The transposition mechanisms of different CAST types vary significantly, with type I-F CAST exhibiting superior integration efficiency and specificity [11]. Rybarski *et al*. identified 1,093 non-redundant type I-F subsystems in metagenomic contigs and suggested that type I-F CASTs were the most common CAST subtype. These putative I-F CASTs exhibit great diversity in both the organization of the *cas* genes and the cargo gene content [7]. Among the type I-F CAST subtypes that were experimentally assessed, significant variation in integration efficiency, optimal integration temperature and protospacer adjacent motif (PAM) requirement was observed [12]. Thus, characterizing additional CAST types and subtypes will provide additional insights that could be used to facilitate further expansion of the CAST-based gene-editing toolset.

The VchCAST (Tn6677) of *Vibrio cholerae* is the best characterized I-F CAST [5,13, 14]. This system mainly comprises the transposon proteins TnsABC, TniQ and the type I-F3 CRISPR-Cas system (Cascade) as the targeting module, while notably lacking the helicase-nuclease Cas3. This distinguishes it from the type V-K system, which features a nuclease-deficient Cas12. TniQ binds to the Cascade as a head-to-tail homodimer, forming the TniQ–Cascade complex [13,15]. Transposition begins when the TniQ–Cascade complex binds to DNA at the targeted site and slightly alters its conformation [14,16]. Then, TniQ is thought to bind to TnsC, further recruiting the TnsAB transposase complex [17,19]. As with the classical Tn7 transposon, TnsA and TnsB cleave the 5′ and 3′ ends of the transposon, respectively [20], transferring it from the original insertion site to a new site, a process known as cut-and-paste transposition. Mutation in TnsA may cause failure of cleavage at the transposon’s 5′ end, resulting in transposition in the ‘copy-and-paste transposition’ manner, which can also lead to the formation of a cointegrated product [21,22]. Due to its programmability and high editing efficiency, the type I-F3 CAST provides an effective gene insertion tool and has attracted much attention in the field of genome editing [23]. The VchCAST has also been used for precise tuning of gene expression by inserting specific promoters upstream of targeted genes [24]. Recently, multi-subunit Cascade effectors derived from VchCAST and a related *Pseudomonas* CAST were used to achieve double-strand break-free large DNA knock-ins in human cells [11]. Furthermore, the I-F3 CAST has been used for precise genome editing of specific micro-organisms within communities, enabling the tracking of barcoded micro-organisms in natural communities [25]. However, there are also practical limitations in the application of type I-F3 CAST. The use of multi-plasmid genetic systems for large DNA insertion is challenging and inefficient due to the complexity of the multi-subunit Cascade system. Additionally, even in the model organism *Escherichia coli*, the single-plasmid system has limited transformation efficiency owing to its high molecular weight (~10 kb). These limitations have prompted the exploration of the host bacteria’s endogenous I-F3 CASTs as simpler alternatives to the genomic editing tools.

*Shewanella* genomes contain a wide variety of mobile genetic elements (MGEs), endowing the hosts with diverse metabolic potential, including multifunctional electron-accepting capabilities [26,27]. Previous studies of MGEs in *Shewanella* have focused on prophages [28,29], plasmids [30] and genomic islands [31]. The contribution of CASTs to genome diversification and environmental adaptation in *Shewanella* remains unclear. In addition, members of the *Shewanella* genus are known for their potential in bioremediation of organic and heavy metal pollutants. For example, *Shewanella* spp. were reported to be able to utilize diverse electron acceptors, including nitrate, chromium [Cr(VI)], radioactive uranium [U(VI)] and selenite in contaminated environments [32,34]. Hence, precise and high-throughput genetic manipulation tools effective in *Shewanella* are needed to better characterize molecular mechanisms underlying bioremediation as well as to efficiently engineer these bacteria to improve their potential bioremediation functions. Although CRISPR-Cas9 has been used to introduce genetic changes, including point mutations and insertion of small DNA fragments in *Shewanella oneidensis*, the efficiency remains low [35]. Gene insertion into *Shewanella* genomes is achieved mostly through the introduction of recombination plasmids, which also lack efficiency, especially while inserting large or multiple DNA fragments [36,37]. Therefore, understanding the distribution and ecological functions of CASTs in *Shewanella* will not only shed light on their contribution to *Shewanella*’s genomic evolution but also offer a valuable source of potential gene-editing tools for *Shewanella* and other CAST-carrying bacteria.

In this study, we explored the diversity and function of CASTs in the *Shewanella* genus. Bioinformatic analyses were first performed to profile the distribution, gene organization, phylogenetic relatedness and cargo gene content of type I-F3 CASTs in all available *Shewanella* genomes in national center for biotechnology information (NCBI). Next, *in situ* genome editing using the host-encoded I-F3 CAST was explored in the representative strain *Shewanella* sp. ANA-3. Heterologous editing with this I-F3 CAST variant in *E. coli* was further assessed, which also demonstrated efficient and programmable DNA integration capability.

## Methods

### Identification of type I-F3 CASTs in *Shewanella*

Genomic sequences of all *Shewanella* strains (*n*=602) were downloaded from the NCBI RefSeq Bacteria database as of 4 December 2023. Putative type I-F3 CASTs were searched in these genomes using the Python library opfi [7]. The left and right boundaries of *Shewanella* CASTs were identified using the Python library Tnacity [3]. The attachment site refers to the gene located closest to the right end (RE) near the *tnsA* gene [4,7]. The attachment site and gene organization for each CAST were manually collected.

Then, the complete sequences of the CASTs were obtained, and the enclosed ORFs were predicted using Prodigal (v2.6.3) under metagenomic mode [38]. The ORF functions were annotated based on the clusters of orthologous genes (COG) database with Diamond (v2.0.5.143) blastp [39]. The antiphage defence systems in these CASTs were detected using DefenseFinder [40]. Annotation of antibiotic resistance genes (ARGs), virulence factor genes (VFGs) and metal resistance genes (MRGs) was based on searches against the comprehensive antibiotic research database [41], virulence factor database [42] and BacMet database [43] using Diamond blastp (--evalue 1e^−5^ --min-score 60).

### Phylogenetic analysis

Phylogenetic analysis of the *Shewanella* CASTs was based on the concatenated sequences of *tnsABC*, *tniQ*, *cas8/5*, *cas7* and *cas6* genes [5]. Phylogenetic analysis of the host strains carrying these CASTs was based on sequences of the housekeeping gene *gyrB* encoding the B subunit of DNA gyrase [44,45]. Sequence alignment was conducted in MEGA-CC [46]. The trees were constructed by FastTree (v2.1.11) [47] and visualized with Chiplot (https://www.chiplot.online).

### Bacterial growth conditions

The strain ANA-3 was grown in 1/2 tryptic soy broth medium at 25 °C, and the *E. coli* strains were grown in Luria–Bertani (LB) medium at 37 °C unless otherwise stated. 2,6-diaminopimelic acid (DAP, 0.3 mM) was added while cultivating *E. coli* WM3064.

### Plasmid construction

All plasmids were constructed using a comprehensive series of procedures. Briefly, PCR fragments with flanking BsmBI restriction sites for cloning were generated using the *ApexHF* HS DNA Polymerase FL (Accurate Biology, China). The PCR products were treated with DpnI restriction enzyme to digest the methylated templates and purified using a gel extraction kit (Megan, China). Subsequently, the fragments were assembled into a complete plasmid using the BsmBI-v2 Golden Gate assembly kit (NEB, USA) following the instructions provided. The assembled plasmids were electroporated into *E. coli* WM3064 or BL21(DE3), depending on the plasmid characteristics. The cloning strains, effector strains and main characteristics of the plasmids used are listed in Table 1. Plasmid sequences are included in Table S1, available in the online Supplementary Material. All PCR primers used are listed in Table S2.

**Table 1.** Characteristics of strains and plasmids used

Backbone	crRNA	Plasmid	Cloning strain			Effector strain
pURR25	crRNA-*pyrF*	**pDonor**	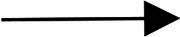 Elec	WM3064	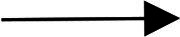 Conj	*Shewanella* sp. ANA-3
pGGAselect	crRNA-*glmS*	**pEffector**	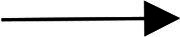 Chem	*Trans2*-Blue	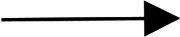 Elec	*lacZ*-Tn-BL21(DE3)
pGGAselect	crRNA-*lacZ*	**pGGA-CAST**	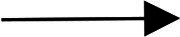 Chem	*Trans2*-Blue	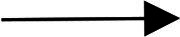 Elec	BL21(DE3) *lacZ*-Tn-BL21(DE3)
pURR25	No	**pURR25-CAST**	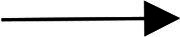 Elec	EC100D *pir+*	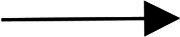 Elec	WM3064
pUC57	crRNA-*lacZ*	**pcrRNA-*lacZ***	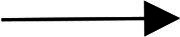	XL10-gold		

The arrow marks the delivery pathway of the plasmid. ‘Elec’ represents electroporation; ‘Conj’ represents conjugation; ‘Chem’ represents Chemical transformation.

The CRISPR array used in this investigation was synthesized by Shanghai Saiheng Biotechnology Co., Ltd. (China). The crRNA-*lacZ* and crRNA-*glmS* were designed according to Klompe *et al*., although they were located on the opposite strand [5]. The spacer sequences and PAMs are listed in Table S1. The 2 kb gene cargo (mini-Tn) used in this study includes the left-end (LE) and RE sequences of I-F3 CAST with a kanamycin resistance gene (*kan^R^*) and a green fluorescent protein gene (*egfp*) in the middle. The LE and RE were cloned from strain ANA-3, and *kan^R^* and *egfp* were cloned from the pEASY-T1 and pURR25 plasmids, respectively [48,49]. The *tnsABC* transposase and the *tniQ-cas8/5*,*7*,*6* Cascade complex genes were cloned from the ANA-3 genome. The pURR25-backbone plasmids are low-copy-number plasmids containing a mobilizable *oriT_IncPα_*, a suicide *oriR_R6Kγ_*, a *lac* promoter, as well as *amp^R^* for screening positive transformants. These are also broad-host-range plasmids that can be shuttled in Gram-negative bacteria [50,51]. In this study, the pURR25-backbone plasmids were electroporated into *E. coli* WM3064 for amplification and then delivered to other strains via conjugation. The pGGAselect from the Golden Gate assembly kit is a high-copy-number plasmid containing a T7 promoter, an origin of replication (*ori*) and a chloramphenicol resistance gene (*cm^R^*), which can be electroporated into *E. coli* BL21(DE3). pUC57 is a commonly used cloning vector carrying an *amp^R^* gene. The pcrRNA-*lacZ* plasmid was constructed using the pUC57 backbone, synthesized by Shanghai Saiheng Biotechnology Co., Ltd. (China), and propagated in *E. coli* XL10-gold.

### Transformation methods

The chemically competent cells of *E. coli* Trans2-Blue were supplied by TransGen Biotech Co., Ltd. (China). The *E. coli* EC100D^TM^ *pir*^+^ electrocompetent cells were from Lucigen (USA). The electrocompetent cells of *E. coli* WM3064 and BL21(DE3) strains were homemade. Following electroporation or heat shock transformation, the *E. coli* cells were incubated in LB at 37 °C for 1 h and then plated on LB-agar plates containing appropriate antibiotics. IPTG was added as the inducer of *lac* and T7 promoters.

For conjugation experiments, the donor strain *E. coli* WM3064 and the recipient strain were grown individually to OD_600nm_ 0.5–0.8. Then, the cells were washed twice with sterile LB medium to remove antibiotics. The donor and recipient cells were mixed in a 1:1 ratio and applied onto 0.45 µm membrane filters, which were then placed on LB-agar plates supplemented with DAP. When *E. coli* WM3064 carrying the pDonor plasmid was conjugated with ANA-3, uracil (50 µg ml^−1^) was additionally supplemented. The plates were incubated at 28 °C for 24–48 h. The cells were resuspended from the membrane filters and plated on selective 1/2 tryptic soy agar (TSA) plates. Positive transconjugants were obtained for PCR confirmation and Sanger sequencing.

### Endogenous transposon assays

The *pyrF* gene was selected as the target site for assessing the endogenous transposition function of ANA-3-CAST, which encodes the orotidine-5′-phosphate decarboxylase. The wild-type strain possessing this gene can convert 5-fluoroorotic acid (5-FOA) into 5-fluorouridine monophosphate (5-FUMP), a toxic compound that leads to cell death; the ∆*pyrF* mutant lacks the ability to convert 5-FOA to 5-FUMP but is also unable to synthesize uracil and must grow in uracil-supplemented media [52]. Therefore, uracil- and 5-FOA-containing medium was used for screening and selection of isolates with integration at the *pyrF* locus, respectively.

The pDonor plasmid was constructed to include a mini-Tn containing two cargo genes (*kan^R^* and *gfp*) flanked by LE/RE transposon ends and a minimal CRISPR array (Repeat-Spacer-Repeat) targeting the *pyrF* gene. To determine whether the mini-Tn could be integrated into the *pyrF* gene via the host’s I-F3 CAST system, *E. coli* WM3064 harbouring the pDonor plasmid was conjugated with the ANA-3 strain. The transconjugants were then plated on 1/2 TSA medium containing uracil (50 µg ml^−1^) and 5-FOA (1 mg ml^−1^) and incubated at 25 °C for 24 h. After incubation, the colonies were scraped off the plates for DNA extraction and PCR analysis. To further obtain individual isolates, the transconjugants were plated on 1/2 TSA plates containing 50 µg ml^−1^ kanamycin, 50 µg ml^−1^ uracil and 0.1 mM IPTG. After incubation for 24 h, colonies were picked for PCR analysis.

### Heterologous transposon assays

To evaluate the transposition capability of the ANA-3-CAST in a heterologous host, the transposition experiment was performed in *E. coli* BL21(DE3) using a pGGA-CAST plasmid. This plasmid harbours the *tnsABC* and *tniQ-cas8/5*,*7*,*6* genes from ANA-3, along with a mini-Tn and a minimal CRISPR array targeting the *lacZ* gene. The experiment aimed to verify whether the CAST system could integrate the mini-Tn into the *lacZ* gene under the guidance of crRNA-*lacZ*. First, the pGGA-CAST plasmid was amplified in *E. coli* 2-Blue and subsequently introduced into BL21(DE3) via electroporation. As a negative control, a non-targeting control plasmid (pGGA-CAST-nt) was constructed according to Klompe *et al*. [5]. Next, the electroporation products were directly spread onto LB-agar plates containing kanamycin and IPTG, followed by overnight incubation at 37 °C. However, likely due to the toxicity of IPTG, this procedure yielded low transformation efficiency with only 24 colonies. In order to better assess the integration efficiency, stepwise screening and induction were further conducted according to the approaches by Klompe *et al*. [5] and Vo *et al*. [53]. Specifically, the electroporation product was evenly spread onto LB-agar plates supplemented with chloramphenicol and incubated at 37 °C overnight. The pooled transformants were spread onto LB-agar plates supplemented with kanamycin, IPTG and X-gal, followed by overnight incubation at 37 °C. The integration efficiency at the *lacZ* locus was calculated based on the counts of blue and white colonies. Next, all colonies were scraped from the blue–white screening plates, and genomic DNA was extracted using the *EasyPure*^®^ Bacteria Genomic DNA Kit for subsequent PCR analysis.

### PCR analysis of transposition

For colony PCR, positive transconjugants were picked and resuspended in liquid medium and lysed at 95 °C for 15 min. The lysate was cooled to room temperature for subsequent PCR analysis. PCR was performed using *EasyTaq* DNA Polymerase (TransGen Biotech, China) in a 25 µl reaction containing 2 µl of lysate supernatant, 12.5 µl of 2×EasyTaq PCR SuperMix and 0.25 µM primers. The thermocycling protocol was set according to the instructions of the reagent kit. Detection was carried out using 1% agarose gel electrophoresis, and PCR products were sequenced by Sanger sequencing.

### PCR analysis of multiple insertions

After the mini-Tn was integrated into the *lacZ* locus of *E. coli* BL21(DE3) by the pGGA-CAST plasmid, PCR was carried out to ensure the pGGA-CAST plasmid had been cured. One pre-integrated BL21(DE3) colony was chosen randomly for preparation of electrocompetent cells, which were then transformed with the pGGA-CAST plasmid again to assess repeated insertion events. Positive transformants were selected on LB-agar plates supplemented with chloramphenicol, which were subsequently induced with IPTG in liquid LB medium. Then, genomic DNA was extracted from these cultures and analysed by PCR to determine whether the mini-Tn was repeatedly inserted at the *lacZ* locus.

### qRT-PCR analysis of SOS response- and CAST-associated gene expression

When fresh inoculum of ANA-3 reached OD_600nm_ ~0.2, mitomycin C (MMC, 1 µg ml^−1^ final concentration) was added to induce the SOS response (DNA repair system) [54], and the cells were shaken for an additional 3 h. Total RNA was extracted using the TransZol Up Plus RNA Kit (TransGen Biotech, China), followed by reverse transcription to cDNA using the HiScript II QRT SuperMix (Vazyme Biotech, China). The resulting cDNA was used as the quantitative reverse transcription PCR (qRT-PCR) template to quantify the expression of SOS-related genes (*recA*, *lexA* and *recN*) [55] as well as the *tnsB* and *cas8* genes. Primer pairs were designed to amplify 98–160 bp fragments for these target genes and two genomic reference genes (*mdh* and *rpoA*) [56,57] (Table S3). qRT-PCR reactions were conducted on the CFX96 Touch Real-Time PCR Detection System (Bio-Rad, USA) using the SYBR Green Pro Taq HS Premix (Accurate Biology, China) with the following amplification conditions: 2 min at 95 °C, 40 cycles of 5 s at 95 °C and 15 s at 60 °C, followed by a melting curve. For each sample, the relative expression levels of target genes were calculated using the 2^-ΔΔ*Ct*^ method for each gene [58]. Three replicates were set for each treatment. The cells grown in the absence of MMC were used as the reference, and *P* values were derived from T-tests, with *P*<0.05 considered statistically significant.

### PCR and qPCR analysis of re-mobilization

To investigate whether the mini-Tn integrated at the *lacZ* locus in *E. coli* BL21(DE3) could be re-mobilized, a pEffector plasmid carrying the TnsABC–QCascade complex genes and a *glmS*-targeting CRISPR array, while lacking the mini-Tn, was constructed. The pEffector plasmid was introduced into the pre-integrated BL21(DE3) colony harbouring a mini-Tn at the *lacZ* site. Positive transformants resistant to chloramphenicol were selected and then induced with IPTG in liquid LB medium to activate the CAST system. DNA was extracted from the cultures, and PCR was carried out to detect whether mini-Tn had integrated at the *glmS* site. Since insertion of mini-Tn at the *glmS* locus with T-LR orientation was not detected by PCR, the primers targeting both ends of mini-Tn in T-RL orientation at the *glmS* locus were designed for qPCR analysis. According to Klompe *et al*. [5], the single-copy gene *ssrA* in *E. coli* was used as the reference gene for normalization purposes. The re-mobilization efficiency (%) for each end is calculated as 100×(2^Δ*Cq*), where Δ*Cq* is the *Cq* (reference gene)−*Cq* (T-RL fragment). The average value of two ends was considered the final re-mobilization efficiency.

### Construction and functional validation of the pURR25-CAST plasmid

The pURR25-CAST plasmid was designed to contain TnsABC and QCascade genes with the *lac* promoter, mini-Tn (*egfp* and *kan^R^*) and restriction enzyme sites and was propagated in *E. coli* WM3064. A helper plasmid, pcrRNA-*lacZ*, carrying a CRISPR array targeting the *E. coli lacZ* gene was also constructed and propagated in *E. coli* XL10-gold. To validate whether the pURR25-CAST plasmid could integrate into the *lacZ* locus as guided by pcrRNA-*lacZ*, the pURR25-CAST was introduced into *E. coli* XL10-gold harbouring the pcrRNA-*lacZ* via conjugation. Transconjugants were selected on LB-agar medium supplemented with kanamycin, ampicillin and IPTG. Colonies were picked from the selective plates, and PCR analysis was conducted to confirm the presence and correct integration of the mini-Tn.

## RESULTS

### Distribution of type I-F3 CASTs in the *Shewanella* genus

**Fig. 1.**
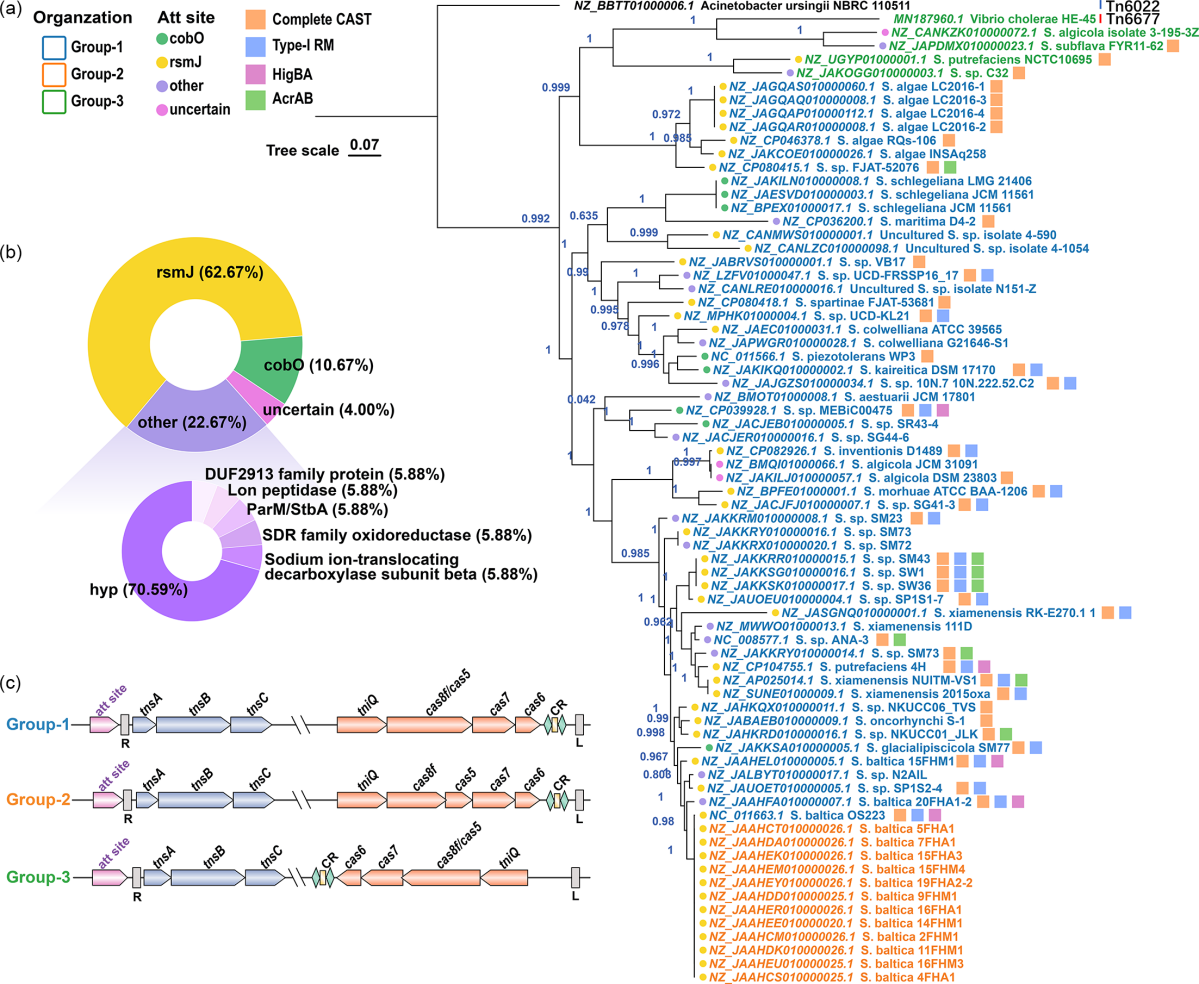
Distribution of type I-F3 CASTs in *Shewanella*. (a) The *tnsABC-tniQ-cas8/5*,*7*,*6*-based maximum likelihood tree of 72 *Shewanella* I-F3 CASTs. The colour of each leaf name corresponds to the gene organization pattern. The colour of the circle before each leaf name indicates the attachment site. The coloured squares after leaf names represent the following: complete CAST sequence obtained (orange), presence of type-I RM system (type-I RM) (blue), HigBA toxin–antitoxin system (rose red) and multidrug resistance genes (*acrAB*) (green) in the cargo DNA.** (b) **Proportion of attachment sites. (**c) **Gene organization patterns of *Shewanella* I-F3 CASTs.

Based on an updated dataset of *Shewanella* genomes, a total of 72 Tn7-like elements encoding the type I-F3 CRISPR-Cas system were identified, covering 12% of the 602 total genomes analysed. I-F3 CASTs were classified into I-F3a and I-F3b types, which utilize different Xre family transcriptional regulators as described by Petassi *et al*. [4]. The Xre regulators of all annotated *Shewanella* I-F3 CASTs clustered with C.Csp231I, which belongs to the I-F3b subtype (Fig. S1). Analysis of insertion sites revealed that 44 *Shewanella* I-F3 CASTs were located in the non-coding region at the 3′ end of the *rsmJ* gene, with a few integrated near *cobO* or other loci (Table S4, Fig. 1b).

The *Shewanella* I-F3 CASTs generally consist of the Tns operon (*tnsABC*), the QCascade operon (*tniQ-cas8/5*,*7*,*6-*CRISPR array) and cargo DNA of typically 10–40 kb, excluding the Tns- and QCascade-encoding regions. In a few strains, the cargo DNA reached as long as 100 kb. *Shewanella* I-F3 CASTs exhibited three gene organization patterns (Fig. 1c): group-1 (*tnsA-tnsB-tnsC-tniQ-cas8/cas5-cas7-cas6-*CRISPR array), group-2 (*tnsA-tnsB-tnsC-tniQ-cas8-cas5-cas7-cas6-*CRISPR array) and group-3 (*tnsA-tnsB-tnsC-*CRISPR array*-cas6-cas7-cas8/cas5-tniQ*) (Note that these classifications are specific to this study). The Tns and QCascade gene operons share the same orientation in group-1 and group-2 but are oppositely oriented in group-3. A CAST tree based on the major CAST genes (*tnsABC-tniQ-cas8/5*,*7*,*6*) was constructed (Fig. 1a) and revealed that group-3 CASTs formed a monophyletic branch together with VchCAST, even though they were distributed in distantly related host strains (Fig. S2), suggesting horizontal transfer of these CASTs. In comparison, the group-2 CASTs were associated with closely related *Shewanella baltica* hosts in a single cluster with over 98% identity in *gyrB* sequence (Fig. S2). Interestingly, the sequences of all group-2 *tnsABC-tniQ-cas8/5*,*7*,*6* genes were identical and differed from that of a group-1 CAST from *S. baltica* OS223 by only one base. This base change resulted in the formation of a start codon, which separates the *cas8/cas5* fusion gene to split the *cas8* and *cas5* genes. These findings together suggest that group-2 CASTs may have originated from group-1 by a point mutation in the *cas8/cas5* fusion gene in their recent ancestor host (Fig. 1a).

### Functional diversity of cargo genes in *Shewanella* I-F3 CASTs

**Fig. 2.**
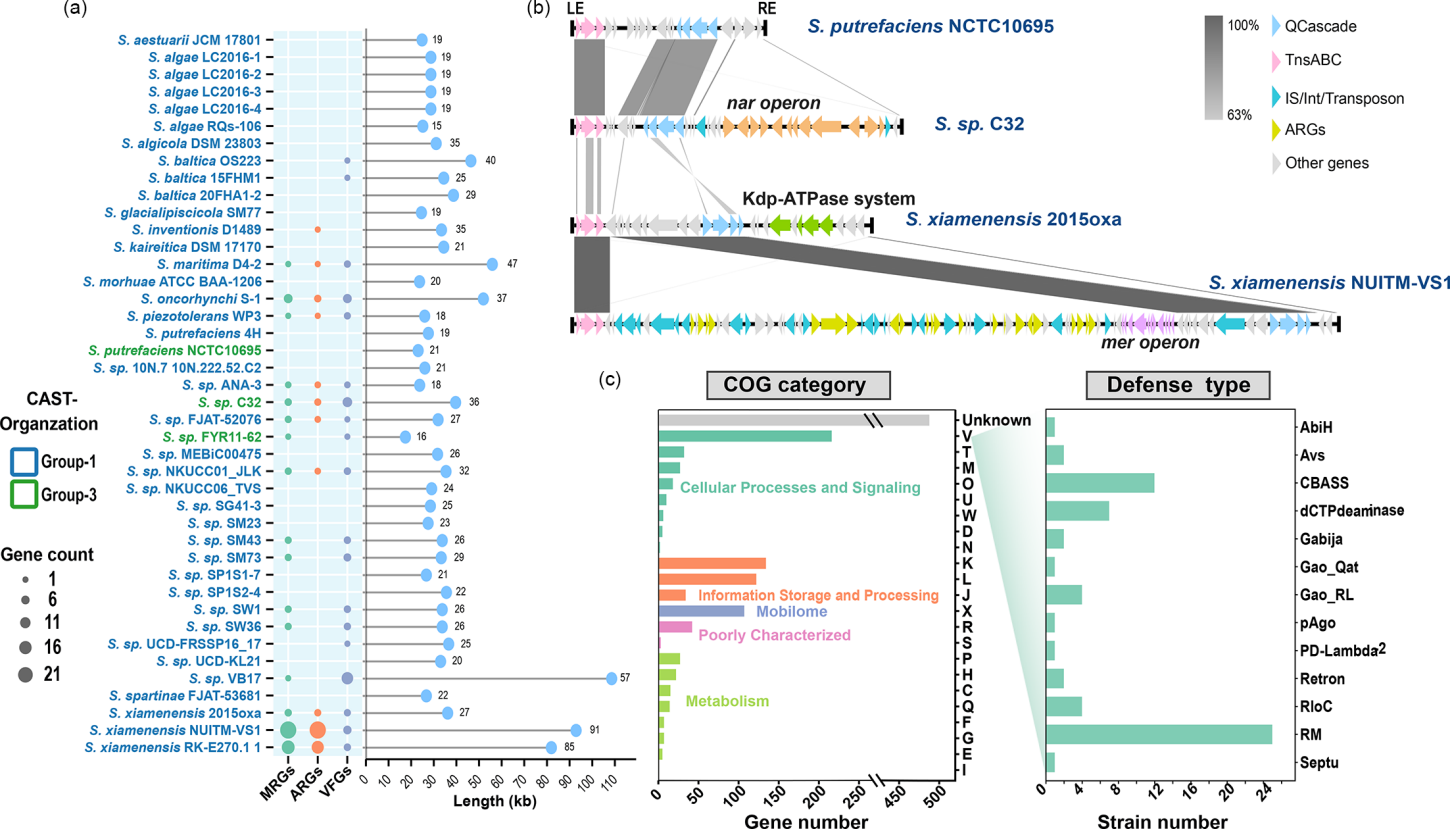
Functional diversity of CAST-associated cargo genes in *Shewanella*. (a) The bubble chart on the left shows the numbers of ARGs, MRGs and VFGs carried by the *Shewanella* CASTs, with the sizes of the circles representing gene counts; the lollipop chart on the right shows the length of each CAST, with the numbers indicating total gene counts. **(b) **Synteny comparison of the complete sequences of four representative CASTs, with the latter three carrying distinct cargo genes. Arrows indicate the locations of coding sequences, and shaded lines indicate the level of homology between pairs of CAST genes. Arrow colours represent different gene components in the CASTs. **(c) **The numbers of cargo genes in individual COG categories and defence system types. The COG categories with the highest frequencies are ‘defence’ (V), ‘transcription’ (K), ‘replication, recombination and repair’ (L) and ‘mobilome’ (X).

The boundaries of I-F3 CASTs were identified in 42 *Shewanella* genomes, comprising 39 group-1 and 3 group-3 CASTs (Table S4). These CASTs were used to investigate the functional diversity of the enclosed cargo genes. The average length of these CASTs was 36 kb (8 genes/10 kb), with the longest reaching 108.6 kb and the maximal cargo gene number exceeding 80 (Fig. 2a). This highlights the potential of I-F3 CASTs in mediating the transfer of large fragments and their role in the hosts’ metabolic versatility.

Over 60% of the cargo genes were classified into COG functional classes, with the highest proportion of defence-related genes (V), followed by those related to transcription (K) and replication, recombination and repair (L) (Fig. 2c-left). A smaller portion of cargo genes were related to amino acid synthesis, nucleotide synthesis, biotin biosynthesis and tRNA modification.

Specifically, *hipB* and *ssl2*, which are involved in DNA transcriptional regulation and replication, recombination and repair, were among the most enriched genes in the *Shewanella* CASTs. Genes encoding a complete type-I restriction-modification (RM) system (HsdR, HsdM and HsdS) were also highly enriched (Fig. S3). This RM system acts as a defence mechanism against foreign DNA and was shared by over half of the CASTs. Additionally, five CASTs were found to carry genes encoding the toxic component of the HigBA toxin–antitoxin system (Fig. S3). This system mediates dormancy or programmed cell death under stressed conditions through differential regulation of the toxin and antitoxin genes. Several antiphage defence systems, such as CBASS, dCTP deaminase, Gao_RL and RloC, were also identified in multiple *Shewanella* CASTs (Fig. 2c-right). Therefore, these CASTs resemble defence islands, potentially safeguarding against foreign DNA damage and cellular stress [12]. Moreover, the *Shewanella* CASTs harbour multiple ARGs, VFGs and MRGs. Notably, the CAST cargo genes of *Shewanella xiamenensis* strains – opportunistic pathogens affecting both humans and animals [59] – contain over 20 ARGs of 9 different types, as well as various VFGs, which may contribute to the antibiotic resistance and virulence potential of the hosts (Figs 2a and S4). Mapping the distribution of several abundant defence systems (type-I RM and HigBA) and multidrug resistance genes (*acrA* and *acrB*) onto the CAST tree further revealed a scattered pattern across different branches, suggesting high mobility of these cargo genes (Fig. 1a).

*Shewanella* I-F3 CASTs carry many complete metabolic gene clusters. For example, *S. xiamenensis* 2015oxa carries genes encoding a high-affinity ATP-dependent Kdp K^+^ transport system (*kdpABC* operon and *kdpD*), which is responsible for transporting K^+^ into prokaryotic cells at extremely low extracellular K^+^ concentrations [60]. *S. xiamenensis* NUITM-VS1 carries a diverse array of ARGs and a broad-spectrum mercury resistance operon (*merTPAR* operon, *merB*, *merD* and *merE*), which transforms and detoxifies both methylmercury (MeHg) and inorganic mercury [Hg(II)] [61]. *Shewanella* sp. C32 carries a complete operon encoding the membrane-bound respiratory nitrate reductase complex (*narGHJI* operon, *narX*, *narL* and *narK*). These findings suggest that I-F3 CASTs may contribute to *Shewanella*’s respiratory versatility and metal-reducing capabilities by facilitating horizontal transfer of genes involved in these processes. Notably, these cargo genes were often flanked by insertion sequences (IS) elements or *intI* integrases (Fig. 2b, c), indicating the role of nested MGEs in the acquisition or exchange of CAST cargo genes [62].

### Endogenous I-F3 CAST enabled efficient genomic integration of DNA fragments

**Fig. 3.**
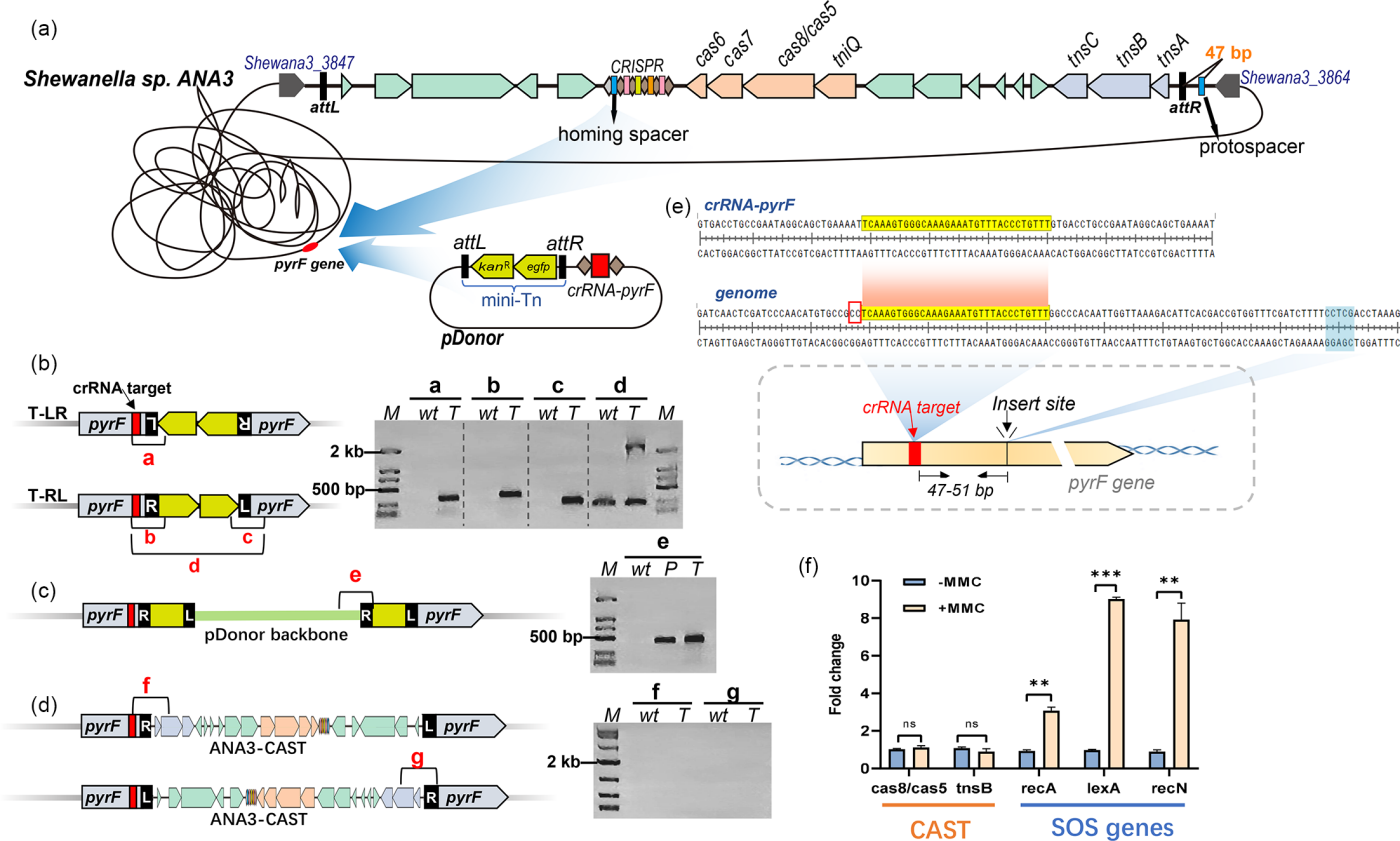
The endogenous I-F3 CAST from *Shewanella* sp. ANA-3 enabled efficient DNA integration. (a) Experimental design: a pDonor plasmid carrying the mini-Tn and the crRNA-*pyrF* cassette was transferred into ANA-3; the mini-Tn is expected to be integrated at the *pyrF* locus by the host-encoded CAST. (**b)–(d) **PCR analysis of transposition, resolved by agarose gel electrophoresis. The letters a–g refer to different primer pairs. Lane ‘wt’ represents the wild-type strain, lane ‘T’ indicates the targeted strain and lane ‘P’ denotes the pDonor plasmid used as a template. Panel B shows the two insertion orientations of mini-Tn at the *pyrF* locus; panel C depicts cointegration; panel D depicts integration of the host-encoded transposon. (**e) **Schematic diagram showing the target site of crRNA-*pyrF* and the insertion site of mini-Tn. (**f) **qRT-PCR quantification of expression of *tnsB*, *cas8/cas5* genes and SOS-related genes (*recA*, *lexA* and *recN*) in ANA-3 treated with MMC. Significance level: ∗∗∗ indicates *P*<0.001; ∗∗ indicates *P*<0.01; ns indicates *P*≥0.05.

*Shewanella* sp. ANA-3 was one of the strains initially identified as harbouring a potential CAST [1]. The ANA-3-CAST was found inserted at the 3′ end of a gene encoding a hypothetical protein (Shewana3_3864) and also flanked the 3′ end of a gene encoding a lipid-binding SYLF domain-containing protein (Shewana3_3847) (Fig. 3a). To validate the programmable DNA integration activity of the endogenous ANA-3-CAST, the pDonor plasmid was conjugated into ANA-3 cells. This plasmid, which is not capable of replicating in *Shewanella* sp. ANA-3, contains a mini-Tn and a minimal CRISPR array targeting the *pyrF* gene located 2.2 Mb away from the nearest native CAST. The conjugation product was spread onto 1/2 TSA plates containing uracil and 5-FOA to select for mutant colonies with integration at the *pyrF* locus. PCR analysis indicated successful integration, and transposition products of both orientations were present (Fig. 3b). PCR was also conducted to examine transfer events of the host-encoded CAST to the *pyrF* locus, yet no positive band was detected (Fig. 3d). Presence of the plasmid backbone was also detected, which likely resulted from plasmid cointegration given that the *oriR_R6Kγ_* replicon could not replicate in ANA-3 (Fig. 3c). To verify cointegration events, 12 colonies were isolated for PCR assessment. Among these, three colonies exhibited cointegration with the pDonor vector backbone (Fig. S5). For all 12 colonies, the insertion site was at 47–51 bp downstream from the protospacer, as confirmed by Sanger sequencing (Figs 3e and S6).

Given the role of the bacterial SOS response in regulating the transfer of various MGEs, its potential impact on CAST activity was investigated. MMC was used to induce the SOS response, and the transcriptional activities of the CAST genes were examined using qRT-PCR. The results revealed increased expression of the SOS-related genes following MMC treatment, yet no significant changes in the expression levels of CAST genes, including *tnsB* and *cas8/cas5*. This suggests a lack of regulatory interaction between the SOS response and the activity of ANA-3-CAST (Fig. 3f).

### Heterologous DNA integration by ANA-3-CAST in *E. coli*

**Fig. 4.**
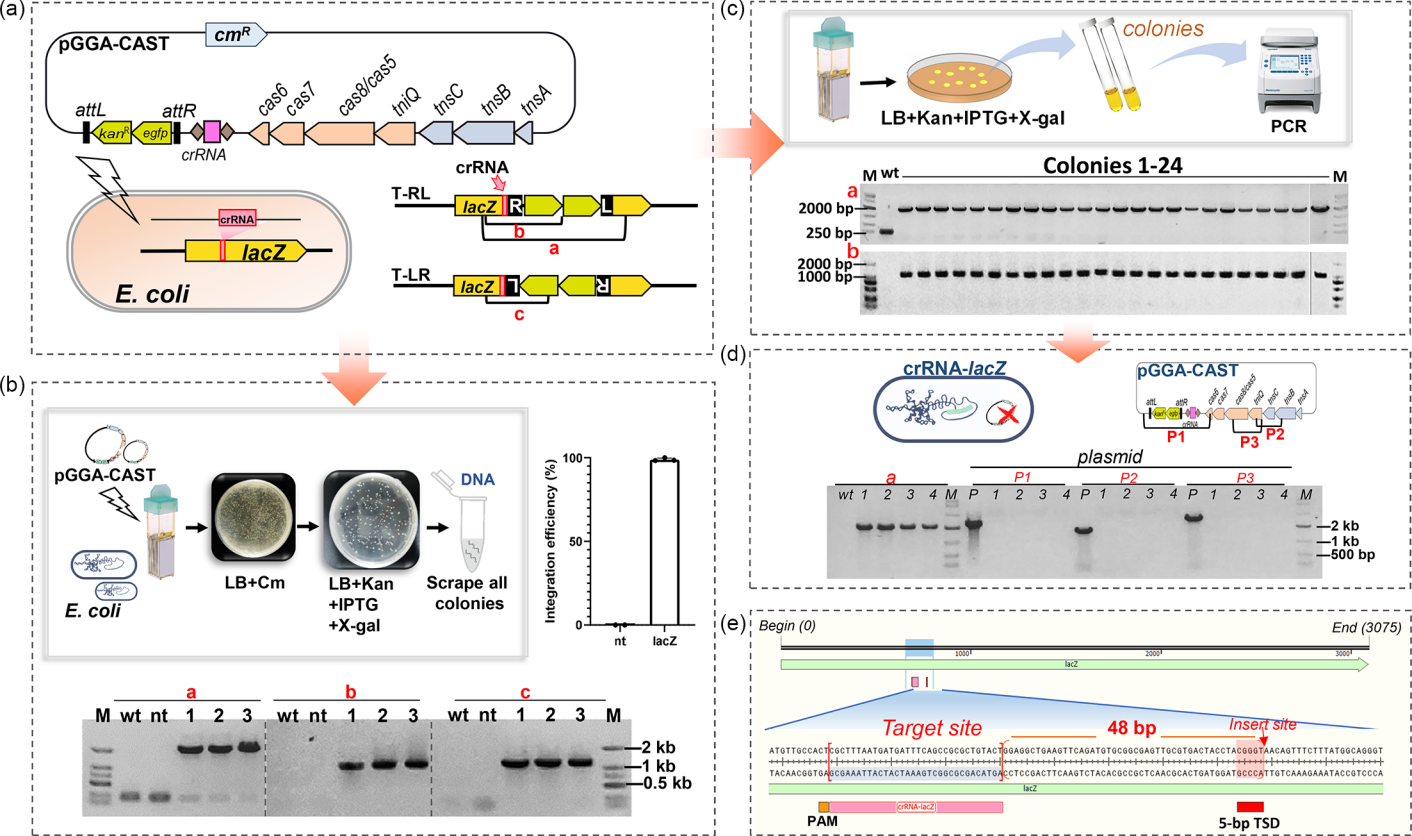
RNA-guided DNA integration with ANA-3-CAST in *E. coli*. (a) Schematic diagram of the single-plasmid CAST (pGGA-CAST) with crRNA-*lacZ* targeting the *E. coli lacZ* gene and PCR primer design for amplification of transposition products in both directions. (**b) **Schematic diagram of a transposition assay protocol. The histogram shows the integration efficiency of mini-Tn at the *lacZ* site based on blue–white screening. The pictures of the plates were taken from the actual experiment. In the blue–white screening plate, the majority of the colonies were white. The bottom panel shows PCR analysis of pooled colonies from three IPTG plates, which indicated that all plates contained integrated products in both orientations (primer pairs a–c). Lane ‘wt’ represents the wild-type strain used as a negative control; lane ‘nt’ represents the negative control treated with non-targeting crRNA. (**c) **Schematics showing a transposition assay protocol. This protocol yielded only 24 colonies in LB-agar plates containing kanamycin and IPTG, all of which were subjected to PCR analysis of transposition product with primer pairs a and b. (**d) **PCR analysis for presence or absence of pGGA-CAST plasmid in four colonies with successful integration of mini-Tn from panel C. P1, P2 and P3 represent different fragments of the plasmid. Lane ‘P’ represents the positive control with the plasmid.** (e) **Schematics showing the insertion site at 48 bp downstream of the target site.

In order to develop the ANA-3-CAST as a heterologous gene-editing tool, the activity of RNA-guided DNA integration was assessed in *E. coli*. The *tnsABC* genes, the *tniQ-cas8/5*,*7*,*6* genes, a 2 kb mini-Tn and crRNA targeting the *E. coli lacZ* gene were all included in pGGA-CAST, a plasmid encoding a simplified ANA-3-CAST (Fig. 4a).

The BL21(DE3) electrocompetent cells transformed with the pGGA-CAST plasmid were directly plated on LB-agar plates containing IPTG and kanamycin (Fig. 4c), which yielded only 24 colonies. PCR analysis revealed that the mini-Tn was integrated into the *lacZ* locus in the T-RL orientation in all colonies, and cointegration with the donor plasmid backbone was not detected (Fig. 4c). Three colonies were randomly selected for Sanger sequencing; the insertion site was located at 48 bp downstream of the target site in all three (Fig. 4e). Notably, the transformants were unable to grow on selection medium containing chloramphenicol even though the *cm^R^* gene was present in the pGGA-CAST plasmid backbone (Fig. 4c). Further evidence that the plasmid backbone was not present after integration was obtained through PCR amplification of three fragments in the plasmid. No amplified products were detected (Fig. 4d), indicating that the pGGA-CAST plasmid was lost and was not repaired by the host’s DNA repair system.

To better assess integration efficiency, IPTG induction was also performed on screened electrotransformants to mitigate the stress and toxicity associated with electroporation and IPTG. Integration efficiency, based on the ratio of white to all colonies (Fig. 4b), was >98%. PCR analysis showed that the mini-Tn was integrated into the *lacZ* locus of *E. coli* in both orientations (Fig. 4b). Based on the results from isolated monoclonal colonies (Fig. 4c), the T-RE insertion orientation appeared to be preferred.

### Target immunity and re-mobilization by ANA-3-CAST in *E. coli*

**Fig. 5.**
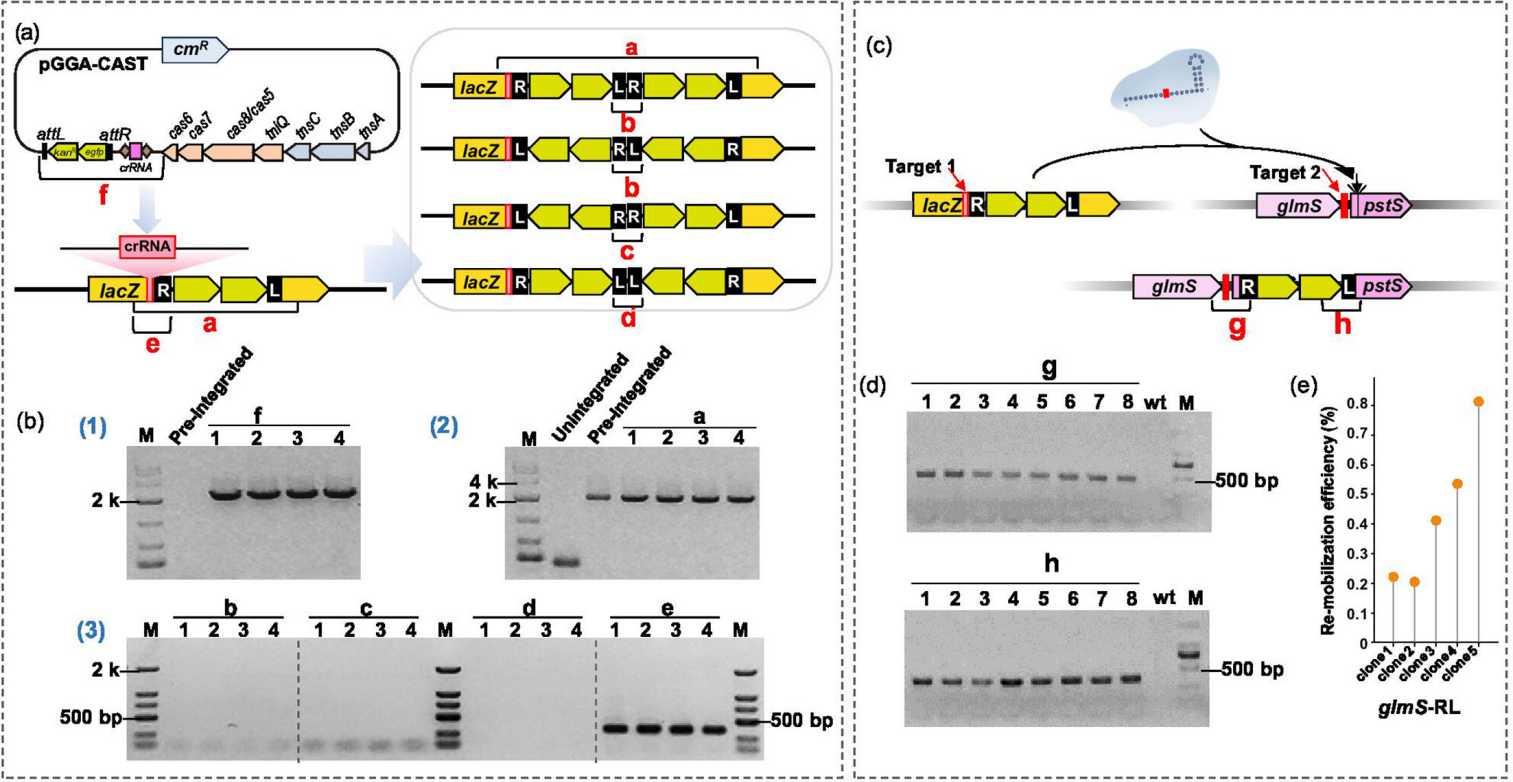
Target immunity and re-mobilization potential by ANA-3-CAST. (a) Schematic diagram illustrating different scenarios of repeated mini-Tn insertion at the *lacZ* locus mediated by pGGA-CAST, along with primer pairs for detecting these re-insertion events (**a–f**). (**b) **Agarose gel electrophoresis analysis of mini-Tn re-insertion. Results in Panel (1) confirmed that the pGGA-CAST plasmid was successfully transformed into cells where integration had previously occurred; Panels (2 and 3) indicate amplification of fragments a–e, suggesting that re-insertion of mini-Tn at the *lacZ* locus did not occur. (**c) **Schematic diagram showing CAST-mediated re-mobilization of mini-Tn from *lacZ* to *glmS* in *E. coli*.** (d) **PCR analysis demonstrating the integration of mini-Tn at the *glmS* locus. Lane ‘wt’ represents the wild-type strain. (**e) **The re-mobilization efficiency of mini-Tn to the *glmS* locus.

**Fig. 6.**
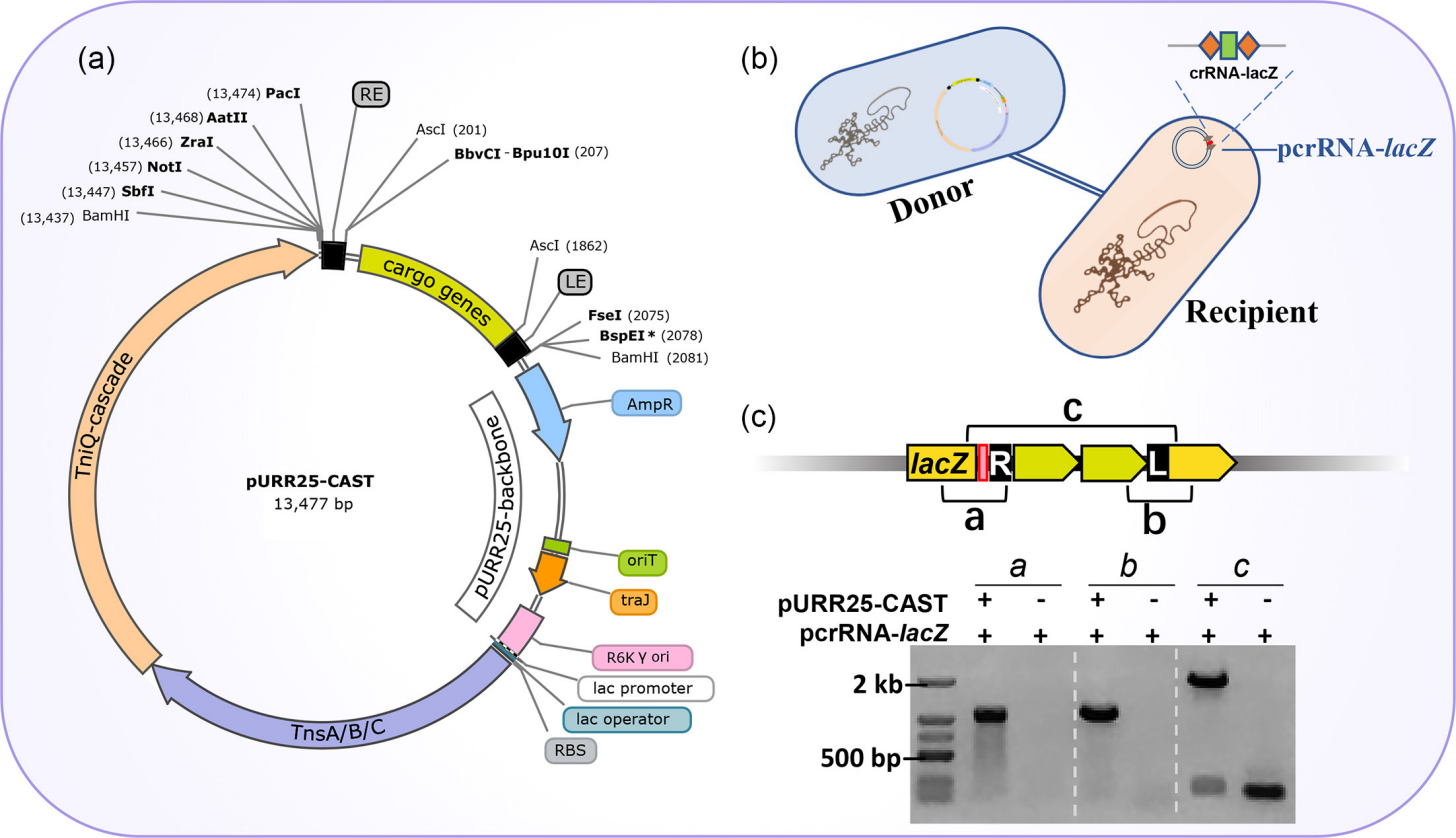
Map and functional validation of the pURR25-CAST plasmid. (**a**) The pURR25-CAST plasmid map. (**b**) Schematic diagram of the conjugation assay for transferring the pURR25-CAST plasmid from the donor strain to the recipient strain. (**c**) Integration of mini-Tn into the *E. coli lacZ* locus, mediated by the pURR25-CAST plasmid.

Some transposons exhibit target immunity, a mechanism that prevents repeated transposition to the same genomic region [63]. Repeated insertion of mini-Tn at the same locus was assessed through PCR. After re-introducing the pGGA-CAST plasmid into the pre-integrated colony, PCR was conducted targeting the entire mini-Tn (Fig. 5a, product a) to screen for the presence of short fragments resulting from four scenarios of re-insertion events (Fig. 5a, products b–d). As a result, re-insertion was not observed [Fig. 5b, (2-3)], while the plasmid remained free and present [Fig. 6b, (1)], indicating potential target immunity conferred by the ANA-3-CAST.

To investigate the re-mobilization capability of the mini-Tn already inserted, a pEffector plasmid without mini-Tn was designed to target the *glmS* gene located 3 Mb away from the *lacZ* gene. PCR analysis indicated re-mobilization of mini-Tn to the *glmS* locus (Fig. 5d). qPCR analysis of five colonies suggested that ~0.4% of mini-Tn was re-mobilized (Fig. 5e).

### Construction of a universal single-plasmid pURR25-CAST

Finally, we further optimized the pGGA-CAST plasmid to broaden its applicability. The pGGAselect backbone of the pGGA-CAST plasmid was replaced with a broad-host-range shuttle vector, the pURR25 backbone, which can only replicate in bacteria containing *π* proteins. Additionally, the T7 promoter, which specifically binds to T7 RNA polymerase and hence has a restricted host range, was replaced with the *lac* promoter (Fig. 6a). In the modified plasmid, the mini-Tn can be replaced via enzyme digestion according to the researcher’s requirement. The CRISPR array can be inserted directly into the plasmid or supplied via an auxiliary plasmid. The final pURR25-CAST plasmid is suitable for programmable integration of DNA fragments into other bacterial genomes through conjugation (Fig. 6b). To experimentally verify its conjugation and transposition capabilities, the pURR25-CAST plasmid was introduced via conjugation into the *E. coli* XL10-gold strain that harboured the auxiliary plasmid (pcrRNA-*lacZ*) containing a *lacZ-*targeting CRISPR array. As a result, successful integration of mini-Tn at the *lacZ* locus in positive transconjugants was confirmed by PCR analysis (Fig. 6c).

## Discussion

### Diversity of *Shewanella* I-F3 CASTs

A recent metagenomic study indicated that CASTs are more common in microbial genomes than previously expected [7], with the I-F3 subtype being the most abundant. I-F3 CASTs are renowned for their remarkable efficiency and precision in gene-editing operations [5,53]. However, studies exploring the ecological role(s) of I-F3 CASTs are still limited. In this study, I-F3 CASTs were predicted in *Shewanella*, a bacterial genus known for its respiratory versatility and role in environmental remediation. Different patterns of gene organization were identified in *Shewanella* I-F3 CASTs, involving the orientation of the Tns and QCascade operons, as well as fusion or separation of *cas8*/*cas5* genes. These differences may contribute to variations in integration efficiency of the CAST systems. The attachment sites of *Shewanella* I-F3 CASTs not only included the previously reported *rsmJ* site [4] but also new sites such as *cobO*, genes encoding ParM/StbA family proteins and hypothetical proteins. Additionally, target prediction of non-homing spacers in a few strains revealed that *Shewanella* plasmids, prophages and Mu transposons may serve as carriers facilitating transfer of the CASTs (Fig. S7). The heterogeneity and widespread distribution of I-F3 CASTs in *Shewanella* genomes together suggest ongoing diversification and their active role in facilitating horizontal gene transfer.

Prediction of boundary ends of the CASTs allowed characterization of the cargo genes of *Shewanella* I-F3 CASTs, which exhibited high plasticity and mobility. Even within the same species or clade, different cargo genes were carried. These cargo genes include a diverse array of ARGs, MRGs, VFGs and other auxiliary metabolic genes. Interestingly, intact metabolic gene clusters such as the nitrate reduction system, the mercury resistance operon and the Kdp K^+^ transport system were also found. This indicates that the CASTs could play a role in the respiratory versatility and metal detoxification ability of the *Shewanella* strains. Notably, the I-F3 CASTs contain various *intI* integrases and IS elements such as IS26, ISCR1 and IS5. Previous research has shown that nested IS facilitated accumulation of genetic diversity within the Tn7 transposon [62]. Here, the diversity of cargo genes in *Shewanella* I-F3 CASTs was also likely associated with these nested MGEs.

As previously described, I-F3 CASTs carry various antiphage defence systems, including CBASS, RM, Retrons and dCTP deaminases [12]. These systems are present in the I-F3 CASTs of *Shewanella* as well, supporting the notion that CASTs function as defensive islands. Particularly, over half of the CASTs contain an intact type-I RM system, which recognizes and cleaves non-self-DNA by methylating self-DNA [64]. This type-I RM system has also been found in the CASTs of *Proteobacteria*, *Actinobacteria*, *Firmicutes*, *Cyanobacteria*, *Chloroflexi*, *Spirochaetes* and *Bacteroides* [3]. Previous research suggested that RM systems in bacterial genomes are primarily carried in the chromosomes in the form of complete RM systems or isolated REase- or MTase-encoding genes [65]. Isolated and complete RM systems may be transferred through different MGEs and/or other transfer mechanisms, with isolated RM systems primarily transferred by phages and conjugative elements [65]. Here, our results support that horizontal transfer of complete type-I RM systems could be mediated by I-F3 CASTs.

### Potential of endogenous and heterologous gene editing by ANA-3-CAST

The type I-F3 CAST is widely distributed in *Shewanella*, suggesting that it may provide a valuable toolset for convenient gene insertion. This study represents the first attempt to explore the RNA-guided DNA integration capability of an endogenous CAST. For the ANA-3-CAST host strain, targeted DNA integration can be achieved by introducing a small pDonor plasmid containing a ‘Repeat-Spacer-Repeat’ cassette and a mini-Tn consisting of *kan^R^* and *gfp* genes flanked by LE and RE. This approach allows precise and effective insertion of sequences into the genomes of non-model bacteria carrying the I-F3 CAST, without the need to introduce the large Tns–QCascade expression system, multi-plasmid systems or additional plasmid curing operations. In this study, mobilization of the host-encoded transposon was not detected. This may be attributed to preferential transfer of the relatively shorter mini-Tn from the plasmid [5]. It is also possible that the transposase had caused DNA breaks via the cut-and-paste transposition mechanism and resulted in cell death after transference of the host-encoded transposon.

This study demonstrated that the endogenous ANA-3-CAST exhibited high transposition activity in mobilizing the mini-Tn from the supplied plasmid. However, the regulatory mechanisms of I-F3 CASTs remain unclear. The SOS response, triggered by DNA damage [66], is an important regulator of the mobilization and transference of many MGEs, such as integrons [67], prophages [68], integrative and conjugative elements [69] and transposons Tn1, Tn5 and Tn10 [70]. However, this study found that the activity of ANA-3-CAST transposase is not regulated by the host’s SOS response, consistent with previous findings about Tn7 [71]. Recent research has shown that the Xre family transcription factor plays an important role in transcriptional regulation of type I-F3 CASTs [4], and genes encoding this factor are also commonly present in *Shewanella* I-F3 CASTs. Additionally, regulatory roles were reported for the integration host factor, which can enhance integration activity of VchCAST [72]. Future research into regulatory mechanisms would facilitate more effective engineering of I-F3 CASTs as DNA integration tools.

ANA-3-CAST also provides an effective, precise and convenient tool for heterologous DNA knock-in. After being assembled into a single-plasmid gene-editing system, an integration efficiency of 98% was achieved in *E. coli*. In addition, the broad-host-range pURR25-CAST plasmid developed in this study further broadens its application across more diverse bacteria. By employing a suicide vector, genomic integration can be achieved without the retainment of extra plasmids, reducing the metabolic burden and genetic perturbation in engineered strains. Although the mini-Tn used in this study was only 2 kb, based on the length of the native ANA-3-CAST (23 kb), the modified version of ANA-3-CAST might be able to integrate much larger DNA fragments. Similarly, it was previously demonstrated that VchCAST can integrate fragments larger than 10 kb [53] and achieve multicopy chromosomal site integration [73].

### Additional characterization of DNA insertion mechanisms by ANA-3-CAST

In the endogenous experiments, a simple insertion product was formed in most cases. However, cointegration of the pDonor plasmid may occur. Similar phenomena have been observed with the Tn7 transposon [74] and VchCAST [22]. This may result from asynchronous cleavage of the donor plasmid by TnsA and TnsB, with cut-and-paste transposition occurring with low-abundance dimeric donor plasmids or homologous recombination between the insertion product and the cellular pool of free donor plasmids [22]. Compared to endogenous experiments, cointegration with the donor plasmid was not observed during the heterologous experiments. The lack of observed cointegration may be associated with factors related to the plasmid backbone, promoters and/or host regulatory mechanisms. Although the cause of such difference remains unclear, our results indicate that heterologous editing facilitated by the modified version of ANA-3-CAST operates more stably compared to editing with the endogenous CAST.

The flexibility of PAM requirements varies depending on specific CAST subtypes. For example, previous work by Klompe *et al*. suggested that sites with any ‘CN’ PAM could be targeted for transposition by VchCAST with indistinguishable efficiency, yet those with ‘NC’ PAM exhibited significantly reduced transposition efficiencies [5]. In contrast, Tn7016 [12] and Tn7479 [75] exhibited nearly PAM-less activity. VchCAST’s preference for ‘CN’ PAM is closely associated with a highly conserved serine residue (S127) and an asparagine residue (N246) in Cas8 [12]. In comparison, the greater PAM tolerance in Tn7016 may be attributed to the presence of alanine at the same position instead of asparagine [12,14, 15]. In ANA-3, the two PAM-related residues in Cas8 are S130 and A248, according to amino acid alignment and predicted protein structure, which are the same as those in Tn7016 (Fig. S7), suggesting a potential of flexible PAM requirement. In the five naturally occurring spacers within the CRISPR array in ANA-3, the target PAM sequences were ‘CC’ and ‘CA’ (Fig. S7). The heterologous experiment in this study indicated a high integration efficiency (98%) with the PAM ‘CT’. Nonetheless, the potential for flexible PAM requirements by testing additional PAM sequences awaits further experimental assessment.

Target immunity prevents multicopy chromosomal integration, which is essential for accurate genome editing. In endogenous editing by CASTs, target immunity also affects target site selection for DNA insertion. Previous research has shown that the presence of Tn7 end sequences on the *E. coli* chromosome reduced integration frequency by Tn7 in nearby chromosomal regions up to 190 kb away [76]. Hence, the presence of native CAST may limit transposition efficiency at the attachment site and its adjacent region. In the case of VchCAST, a detailed study revealed target immunity with 0–20% relative efficiency at target sites 0–5 kb away but no such effect at target sites that were 1 Mb away [53]. In a different study, multicopy chromosomal integration at the same locus was observed, and its likelihood increased with prolonged incubation time [77]. Here, we did not detect repeated insertion at the same site for ANA-3-CAST. Insertion was successful at the site 2.2 Mb away from the native CAST. Although this study cannot exclude the possibility of rare repeated insertion events as a result of prolonged incubation or potential replacement of the original mini-Tn by a new mini-Tn at the same locus, the substantial reduction in detectable integration events supports the effect of target immunity by ANA-3-CAST. However, further thorough assessment of the conditions affecting target immunity will be necessary for applications that require precise genomic editing.

To achieve a balance between bacterial conjugation efficiency and growth efficiency, the temperature used for integration assays was set at 28 °C. Studies by Roberts *et al*. [75] and Klompe *et al*. [12] showed that integration efficiency in *E. coli* was lower at 37 and 30 °C compared to 25 °C. This temperature dependence on integration efficiency may be associated with the origination of I-F3 CASTs from strains colonizing environments with naturally lower ambient temperatures [75]. Therefore, future research may look into the association between integration efficiency and optimal growth temperature of the host bacterium carrying the CAST.

## Conclusions

This study revealed the presence of I-F3 CASTs in 12% of all *Shewanella* genomes sequenced. These CASTs carry a diverse range of functional genes, including those involved in key metabolic functions such as defence, electron transfer, virulence and antibiotic and metal resistance. These findings underscore the role of I-F3 CAST in promoting genomic plasticity and ecological adaptation of the *Shewanella* genus. Furthermore, this study provided the first demonstration of efficient *in situ* genome editing by an endogenous CAST in the strain *Shewanella* sp. ANA-3. The transposition activity of ANA-3-CAST was also assessed through heterologous expression in *E. coli*, achieving 98% integration efficiency. These findings together highlight the role of I-F3 CAST in promoting genomic diversification of *Shewanella* and their potential as a toolset for efficient genetic manipulation.

## Supplementary material

10.1099/mgen.0.001476 Uncited Supplementary Material 1.

10.1099/mgen.0.001476 Uncited Supplementary Material 2.

## Abbreviations

ARG: antibiotic resistance gene
CAST: CRISPR-associated transposon
COG: clusters of orthologous genes
DAP: 2,6-diaminopimelic acid
5-FOA: 5-fluoroorotic acid
5-FUMP: 5-fluorouridine monophosphate
IS: insertion sequences
LB: Luria–Bertani
LE: left end
MGE: mobile genetic element
MMC: mitomycin C
MRG: metal resistance gene
NCBI: national center for biotechnology information
PAM: protospacer adjacent motif
qRT-PCR: quantitative reverse transcription PCR
RE: right end
RM: restriction-modification
TSA: tryptic soy agar
VFG: virulence factor gene
