# Supplementary material for: Type I-F CRISPR-associated transposons contribute to genomic plasticity in Shewanella and mediate efficient programmable DNA integration
Source: Microb Genom. 2025 Aug 19;11(8):001476. doi: 10.1099/mgen.0.001476 (PMC12452197; doi:10.1099/mgen.0.001476)
Supplement: Uncited Supplementary Material 1. [file mgen-11-01476-s001.pdf]

Supporting Figure

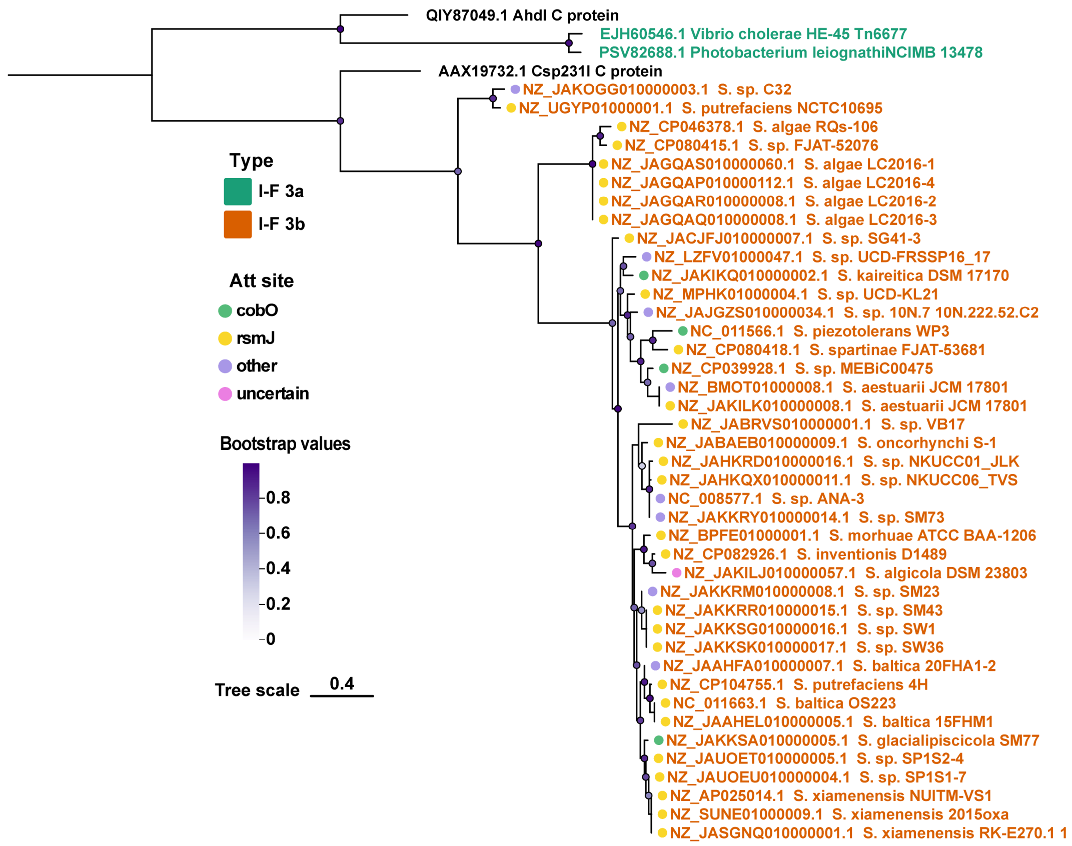

Figure S1. Maximum Likelihood tree of Xre proteins from *Shewanella* I-F3 CASTs. The Xre protein sequences were aligned using MEGA-CC [1], and the tree was constructed with FastTree [2]. The C.AhdI and C.Csp231I were used as representatives for I-F3a and I-F3b Xre proteins [3], respectively. The Xre proteins from all *Shewanella* I-F3 CASTs cluster with C.Csp231I.

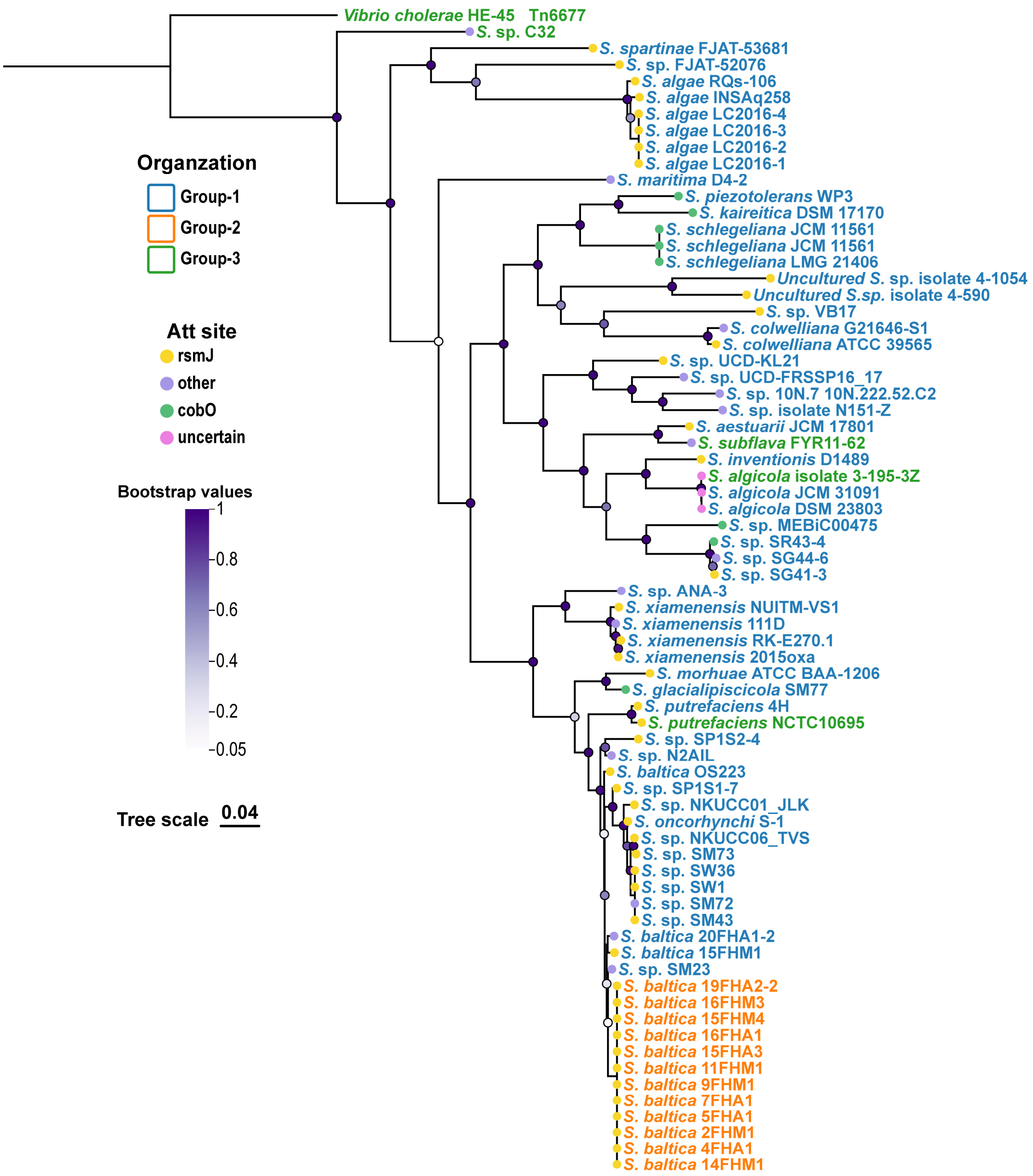

Figure S2. Phylogenetic tree of *Shewanella* I-F3 CAST host strains. The tree was constructed with the maximum likelihood method using the sequences of the housekeeping gene *gyrB*.

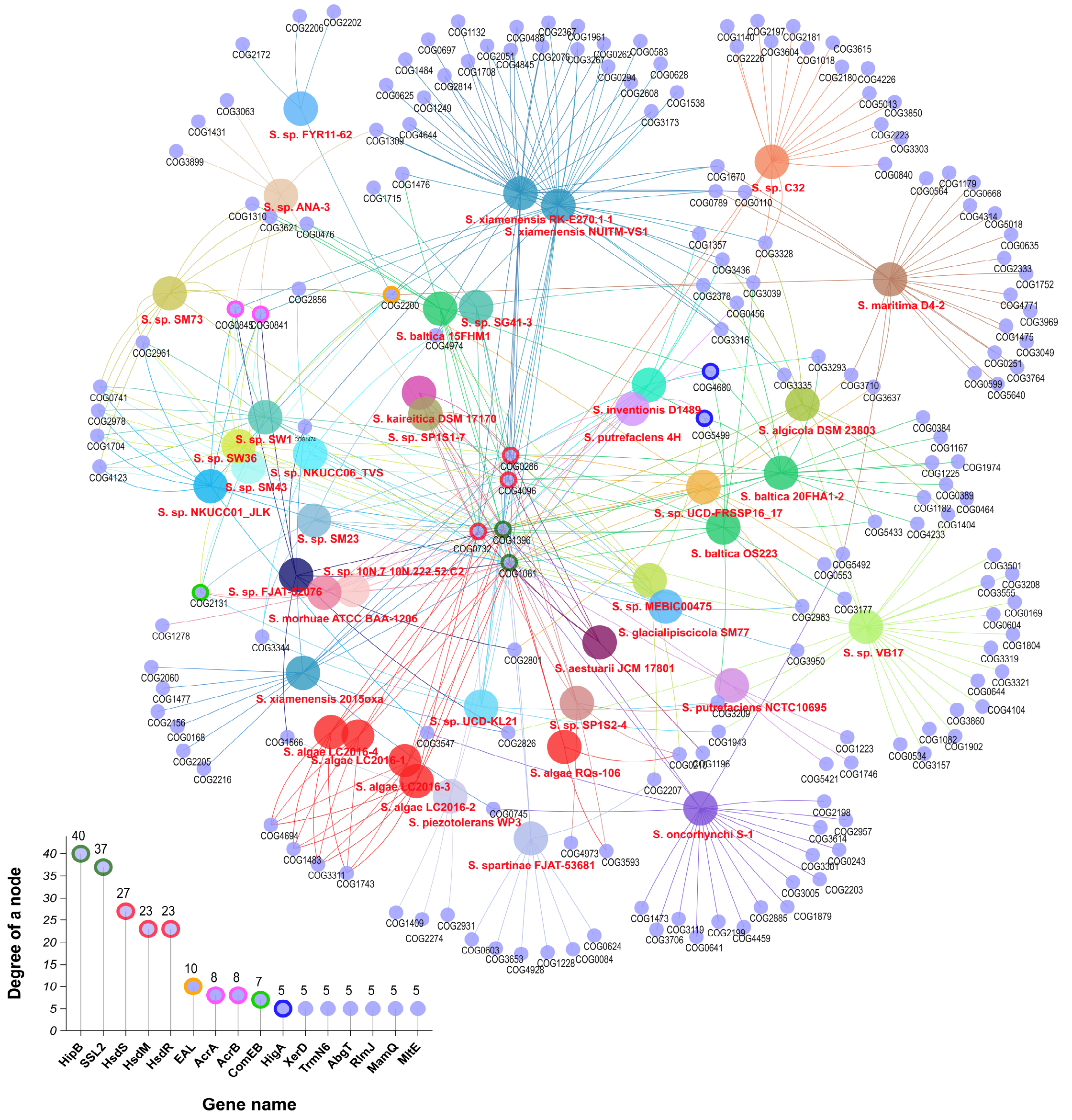

Figure S3. The shared and unique cargo genes carried by different *Shewanella* CASTs. The larger colored circles represent *Shewanella* strains, with the colors representing individual *Shewanella* species. The smaller purple circles represent genes with COG annotations. The lines link the genes with the host bacterial strains, and line colors match the colors of the strains. The bottom left corner shows the degree of 16 nodes representing the most shared genes. The colors of circle outlines correspond to the COG annotations in the network diagram. The network was created using OmicShare Tools (https://www.omicshare.com/).

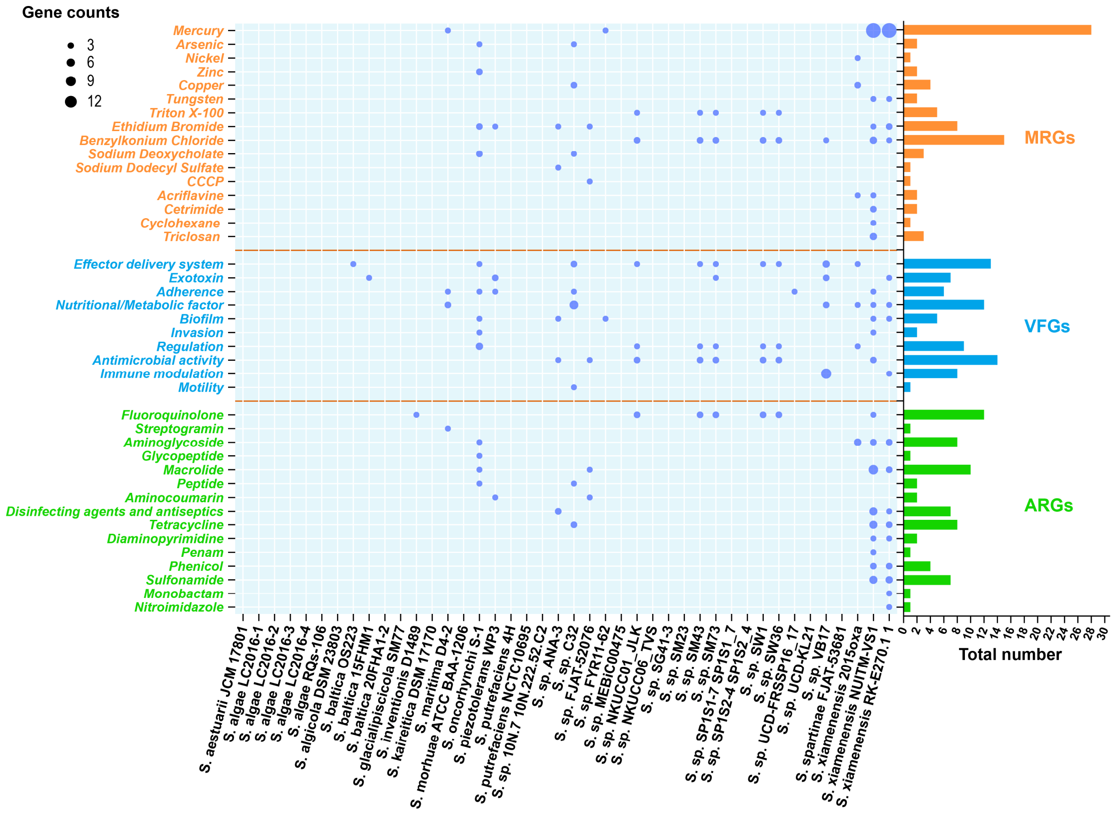

Figure S4. The diversity and numbers of ARGs, MRGs and VFGs in *Shewanella* CASTs. The bubble chart shows the distribution of ARGs, MRGs and VFGs categories. The size of the circle indicates the gene number of each gene class within the CASTs.

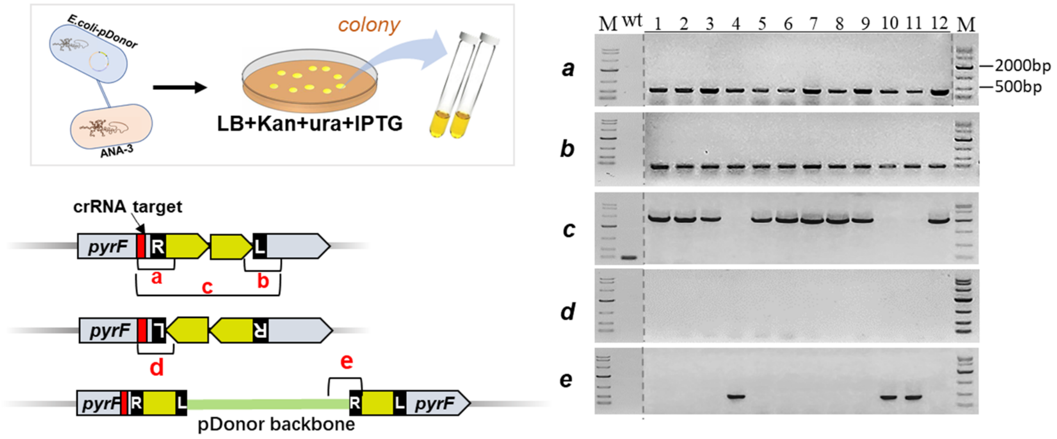

Figure S5. Integration analysis of mini-Tn at the *pyrF* locus mediated by the endogenous I-F3 CAST system based on monoclonal PCR. Twelve distinct clones were obtained after multiple rounds of isolation and purification. Lines 4, 10, and 11

amplified regions a and b, indicating integration at *pyrF*. No fragment was obtained while amplifying the whole mini-Tn, but the e fragment of the plasmid was obtained, suggesting that mini-Tn cointegrated with the pDonor plasmid at the *pyrF* locus.

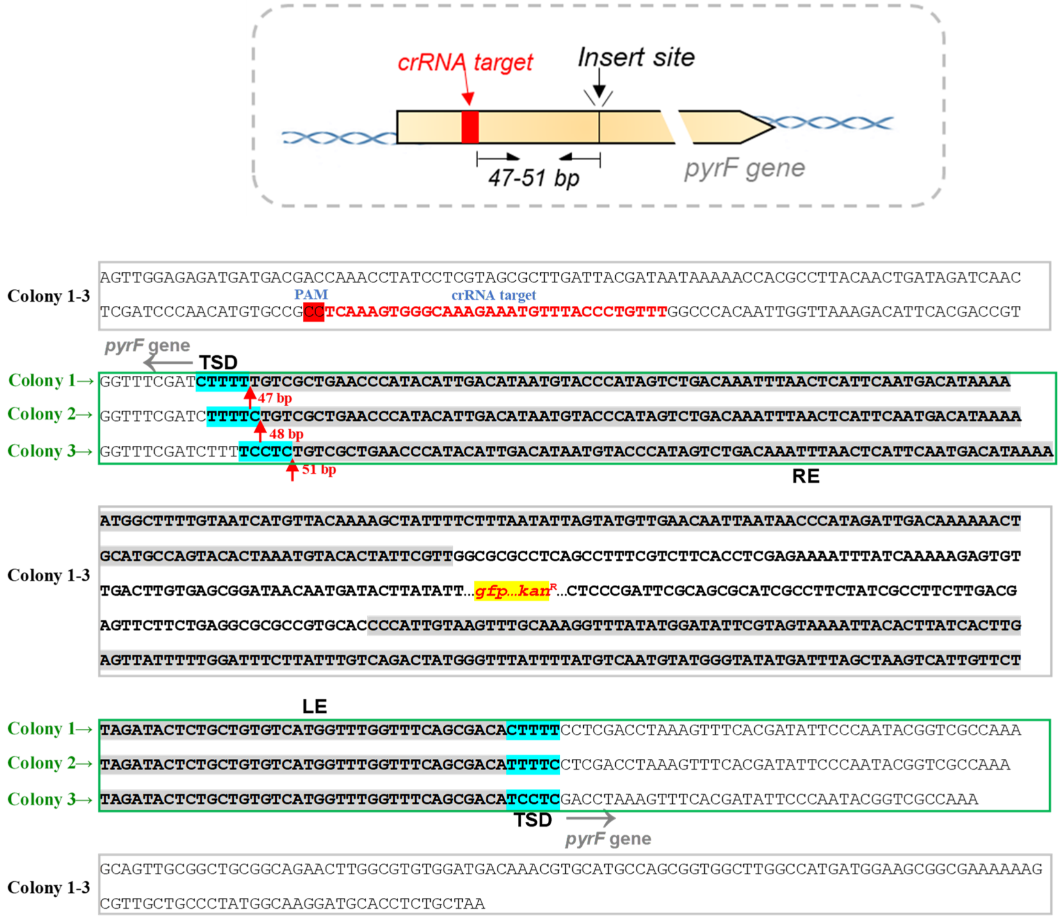

Figure S6. Schematic diagram (top) and sequences (down) of mini-Tn integration into the *pyrF* gene in three ANA-3 single colonies. The grey shading represents the left end (LE) and right end (RE) of the mini-Tn transposon, respectively; the red arrow indicates the insertion site, and the blue highlight indicates the 5 bp target site duplication (TSD) formed after insertion. The sequences at the insertion sites of the three colonies are shown separately (green boxes), while the rest of the sequences (grey boxes) are identical. The sequences between LE and RE are kan^R and *egfp* genes.

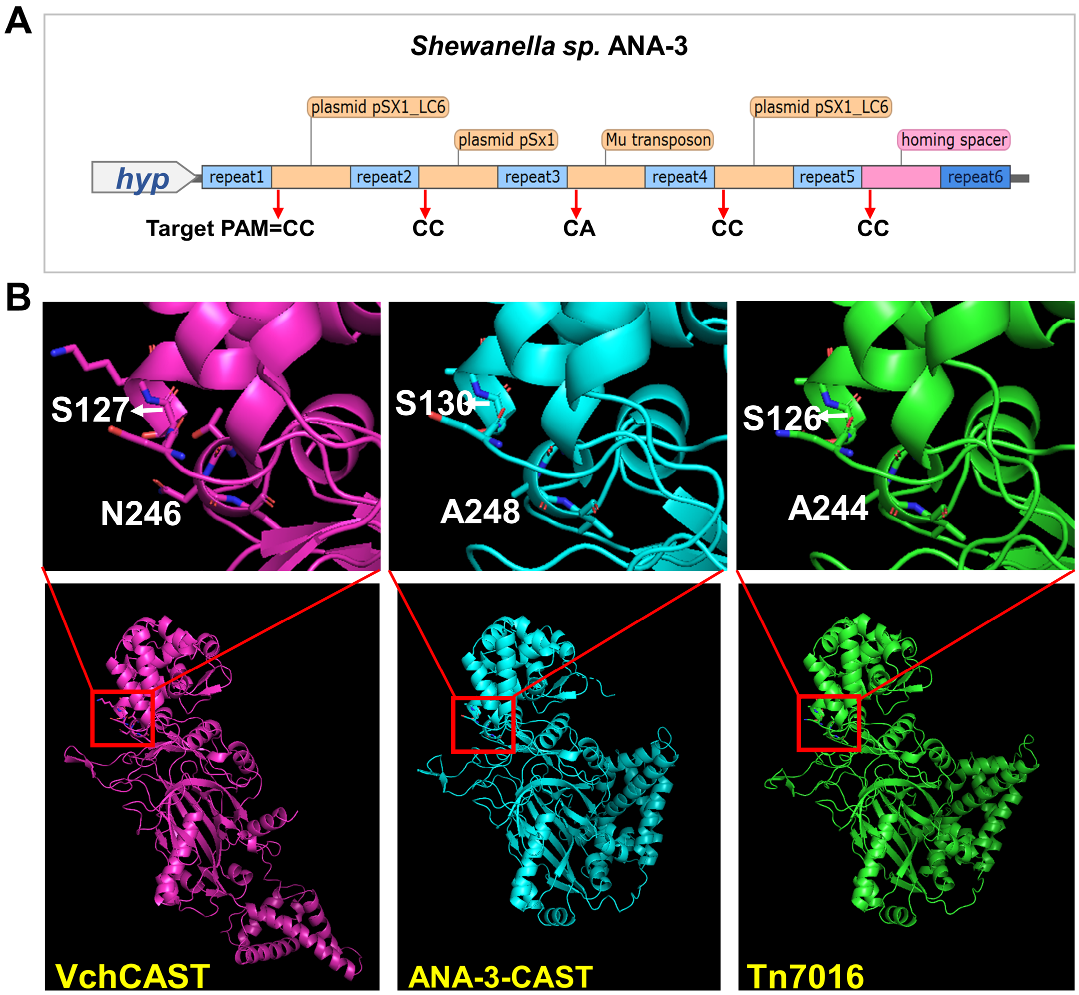

Figure S7. (A) The PAMs and targeted loci of ANA-3-CAST. Blue represents 'repeat' sequences, dark blue indicates 'atypical repeat' sequences, orange denotes 'spacer' sequences, and magenta represents 'homing spacer' sequence. **(B)** The two PAM-related residues of Cas8 proteins in VchCAST, Tn7016 and ANA-3-CAST. ANA-3-CAST exhibits a protein structure similar to Tn7016, but different from VchCAST. The protein structures were modeled using AlphaFold3 [4], followed by structural alignment performed with PyMOL [5].

References

1. **Kumar S, Stecher G, Peterson D, Tamura K**. MEGA-CC: computing core of molecular evolutionary genetics analysis program for automated and iterative data analysis. *Bioinformatics* 2012;28(20):2685-2686.

2. **Price MN, Dehal PS, Arkin AP**. FastTree: Computing Large Minimum Evolution Trees with Profiles instead of a Distance Matrix. *Molecular Biology and Evolution* 2009;26(7):1641-1650.

3. **Petassi MT, Hsieh SC, Peters JE.** Guide RNA categorization enables target site choice in Tn7-CRISPR-Cas transposons. *Cell* 2020;183(7):1757-1771.

4. **Abramson J, Adler J, Dunger J, Evans R, Green T et al.** Accurate structure prediction of biomolecular interactions with AlphaFold 3. *Nature* 2024;630(8016):493-500.

5. **Schrödinger L, DeLano W**. PyMOL. *Retrieved from http://wwwpymolorg/pymol* 2020.
